# Obesity perception survey among youth in Turkey: instrument development and test-retest reliability

**DOI:** 10.3906/sag-1811-56

**Published:** 2019-08-08

**Authors:** Wasantha JAYAWARDENE, Salih PINAR, Mohammad TORABI, Pengcheng XUN, Mustafa ÖZER

**Affiliations:** 1 School of Public Health - Bloomington, Indiana University, Bloomington, Indiana USA; 2 Faculty of Sports Science, Marmara University, İstanbul Turkey; 3 Faculty of Sports Science, İstanbul Gedik University, İstanbul Turkey

**Keywords:** Obesity, perception of problems and solutions, adolescents, young adults

## Abstract

**Background/aim:**

We aimed to develop an instrument that can assess the perceptions and opinions of young people regarding the causes and consequences of obesity and the role of individuals, families, communities, and government in addressing obesity.

**Materials and methods:**

A 36-question (101-item) survey was developed by adopting, translating, and revising multiple-choice or Likert-scale questions from existing surveys to assure construct cross-cultural validity. A two-factor mixed-effects model estimated the intraclass correlation coefficient (ICC) to measure the test-retest reliability of questions administered 2 weeks apart to a convenient sample of İstanbul high school and university students, aged 15–25 years (n = 122).

**Results:**

The mean ICC for university and high school was 0.70 and 0.63, respectively. University students were more consistent in relating the problem to society and public policy preferences. High school students were more consistent in relating the problem and solution to themselves and their immediate environments. Using a 0.5 cutoff for the ICC’s lower 95% confidence limit, followed by reevaluation of the question flow, a 19-question (36-item) survey was retained for adolescents and a 26-question (52-item) survey for young adults.

**Conclusion:**

While the survey items have moderate to excellent reliability for high school and university students, it can be administered longitudinally to suggest changes to policies and interventions, and after cross-cultural validation, it can be utilized to compare obesity perceptions across different populations.

## 1. Introduction

Having doubled in more than 70 countries since 1980, an estimated 5% of children (108 million) and 12% of adults (604 million) are currently obese [1]; the prevalence is higher among women [2]. If not controlled, over one billion adults worldwide will be obese by 2030 [3]. Obesity burden is generally higher in developed countries, although the proportional contribution of each underlying cause varies by region, country, and community [2]. Some countries with a high obesity burden and/or an emerging obesity epidemic do not yet acknowledge this critical health problem with its numerous socioeconomic consequences [4]. Individuals are generally considered more responsible than governments for creating solutions, although both environmental and behavioral trends contribute to the emerging obesity pandemic [5–7]. 

While 27.8% of adults in Turkey are obese, 34.1% are overweight. Obesity prevalence is 34.0% among women and 21.7% among men, whereas overweight prevalence is 30.1% among women and 38.0% among men [8]. Straddling Europe and Asia, Turkey has cultural connections to both continents spanning centuries. Accordingly, a mixture of problems from developing and developed countries can presumably contribute to Turkey’s obesity burden, which has been worsening steadily since the 1990s [9]. Everyday consumption of calorie-dense, once-in-a-while festive foods, increased processed food consumption, and decreased physical activity are considered to be the emerging causes. In addition to the healthcare costs of obesity-related chronic diseases, Turkey currently spends $5 billion annually in support of an emerging, lucrative industry of weight-loss products, online support groups, and dietitians.1 Various studies have explored obesity prevalence in Turkish adults [9,10]. 

Numerous surveys have been conducted to evaluate the perceptions of childhood and adult obesity in both developed and developing countries [11–22]. For example, the Obesity Perception Survey of the U.S. (OPSUS), a nationally representative survey of U.S. adults (n = 1011) [23], assessed public perceptions and opinions regarding the causes and consequences of obesity, including links to other chronic conditions. OPSUS also explored the roles of individuals, families, communities, and respective governments in addressing obesity. Similarly, the Obesity Perception Survey of the European Union (OPSEU), conducted in seven countries (n = 14,000), assessed obesity awareness, including its causes, implications, and treatments, plus self-reported weight status. Additionally, a multinational survey assessed obesity perceptions and policy preferences among policymakers in Europe and the Americas [21]. However, no study has evaluated perceptions and beliefs about obesity among youth (i.e. adolescents and young adults) and adults in Turkey, including their preferred solutions.

The variations of obesity-related social norms, perceptions, and preferred solutions across countries, and even across geographical regions, age groups, and social classes within a country, make it challenging to utilize a standard survey for investigating these constructs. This study is part of a research collaboration to assess obesity-related perceptions and preferences in Turkey. This study aimed to develop a survey for adolescents and young adults by translating and revising questions from existing surveys [22,23] to accommodate the Turkish cultural context and subsequently to evaluate the construct validity and test-retest reliability of survey questions administered to İstanbul youth. 

## 2. Materials and methods

The Obesity Perception Survey among Youth in Turkey (OPSYT) was designed for self-administered, standardized data collection from adolescents and young adults in the region. It was pilot-tested in İstanbul, considering its central location in the target region. In 2016, the high school (ages 14–17) net enrollment rate in Turkey was 79% for males and 78% for females [24]. In 2017, about 31% of men and 32% of women in the 25–34 age group had completed some tertiary education [25]. Considering that the target age group is 15–25, the survey was intended to accommodate the expected average literacy level of high school students. The survey structure and a majority of OPSYT questions were based on the OPSUS and OPSEU surveys [22,23], validated with permission, and were subsequently revised as necessary. All the questions were closed-ended and designed as either multiple-choice (single or multiple answers) or Likert-scale questions. The draft OPSYT survey aimed to evaluate the perceptions of obesity-related problems and solutions in both the public sphere (societal level) and the private sphere (the participants and persons known to the participants). Hereafter, ‘problem’ denotes obesity-related problems either in the public or private sphere while ‘solution’ stands for existing or potential answers to such problems in either sphere.

Problem Perception – Public Sphere (‘Problem-Public’), 15 questions: How participants understand and perceive the magnitude of obesity problem compared to other health issues in Turkey, potential causes and consequences of obesity, the relationship between overweight and overall health status, and obesity-related societal discrimination. 

Solution Perception – Public Sphere (‘Solution-Public’), 6 questions: Participants’ opinions regarding potential treatments for obesity, including morbid obesity, and the responsibility of individuals, families, communities, healthcare providers, various institutions, and government for solving the obesity problem of the country along with the participants’ support for existing or hypothetical policies targeting obesity. 

Problem Perception – Private Sphere (‘Problem-Private’), 6 questions: Participants’ overall health status, including height, weight, and perceived body image, plus knowing a person with obesity. 

Solution Perception – Private Sphere (‘Solution-Private’), 5 questions: Instructions received from participants’ healthcare providers about obesity prevention or treatment and participants’ behavioral strategies for weight management plus awareness of physical and social environments that promote or impede healthy behaviors. 

Sociodemographic, 4 questions: Basic information about participants’ sex, age, income, and area of residence. 

Some questions were presented in tabular format to reduce the target response time to 15 min. The questions were listed continuously with no topical dividers or skip patterns. As this study evaluated perceptions, not attitudes, a ‘don’t know’ option was included where relevant (27 of 36 questions) to allow the respondents to indicate no previous consideration of a particular issue. All response choices facilitated coding and data analysis.

### 2.1. Assessment of construct validity 

The survey was originally composed in English and translated into Turkish. To establish face validity, two U.S. experts individually reviewed each question, with response options, to ensure the operationalization of each construct against a detailed description of the relevant content domain [26,27]. Subsequently, two Turkish experts individually reviewed the questions, with response options, to ensure that each met the average literacy level of Turkish high school students and Turkish contextual social acceptability (i.e. cross-cultural validity). The survey was revised accordingly and reassessed by all the experts. Prior to utilization, it was also back-translated to English by a professional translator to ensure fidelity with original concepts.

### 2.2. Assessment of test-retest reliability 

#### 2.2.1. Study population, setting, and design

In İstanbul, there are over 400 schools with approximately 50 high schools and 44 higher education institutions, including eight public universities. The study population consisted of high school students, typically 15–18 years old, with an approximately 1:1 sex-ratio, plus university undergraduates, typically 19–25 years of age, with a slightly higher percentage of males (Table 1). While the main project study will involve a random sample of İstanbul high school and college undergraduate students, this instrument development study was conducted with a convenience sample of public high school and university students during spring 2016. For both study samples, the project aimed for representative sex and age-group distributions. 

**Table 1:** Demographic characteristics self-reported by students* (N = 122).

Variable	Category	High school (N = 46)	University (N = 76)
Sex	Male	47.8%	62.5%
Age	Largest group (%)	17–18 years (82.6%)	21–22 years (36.2%)
	Second largest group (%)	15–16 years (15.2%)	23–24 years (27.6%)
Area	Urban	92.4%	89.5%
	Suburban	5.4%	9.9%
Income	Below the middle	25.0%	15.9%
	Above the middle	44.6%	61.6%
	Don’t know	30.4%	22.5%
Overall health	Excellent	15.2%	21.0%
	Very good	32.6%	44.7%
	Good	40.2%	31.6%
	Fair	12.0%	1.3%
	Poor	0.0%	1.3%
BMI	Mean (standard deviation)	20.9 (3.0)	22.5 (2.6)

* Each cell is the average of two assessments taken 2 weeks apart. BMI: Body mass index.

#### 2.2.2. Procedure 

The survey was administered to each student twice, 2 weeks apart, an interval that was long enough to prevent them from recalling previous answers but short enough to prevent changes in perceptions over time [28,29]. The self-administered survey was distributed at each site by two researchers. Participation was strictly voluntary and the respondents were not incentivized. The data entry template consisted of validation rules for each question and the entry was supervised by a coauthor. The Ethics Review Committee of Marmara University (İstanbul) approved the study.

#### 2.2.3. Statistical analysis

The data were analyzed using STATA-14.0 [30]. Only the results of those respondents who completed both surveys (test and retest) were retained for analysis. For variables with more than 5% missing values, missing value analysis revealed no systematic patterns based on the available sociodemographic variables [31]. Missing values for each variable were replaced with the mean of sex-based variables. Univariate and multivariate outliers were deleted. The survey primarily collected categorical data, so frequencies and percentages were calculated for each categorical variable. Means and standard deviations were calculated for continuous variables. 

The aim was to assess consistency between test and retest scores of individuals (test-retest reliability), but not relative consistency, which compares individuals in the group relative to the others. Therefore, the intraclass correlation coefficient (ICC) with a two-factor mixed-effects model was utilized [32]. In line with the prior literature, ICC < 0.50 was considered ‘poor reliability’, 0.5 ≤ ICC < 0.75 ‘moderate reliability’, 0.75 ≤ ICC < 0.90 ‘good reliability’, and >0.90 ‘excellent reliability’ [29]. The reliability of each item was determined based on the 95% confident interval (95% CI) of the ICC estimate, rather than the ICC estimate itself, considering that the ICC approximation in a test-retest reliability study is not the true ICC but only an expected value of the true ICC. Hence, the lower limit of 95% CI being greater than 0.5 was considered to be the criterion for ensuring test-retest reliability in this study. 

## 3. Results

The questionnaire validation study involved 122 students with repeated measures; 46 of them were high school students (47.8% males) and 76 were university students (62.5% males). In the school sample, 83% of students were 17–18 years old, and in the university sample, 85% were between 19 and 24 years of age (Table 1). According to self-reported weight and height, the mean body mass index (BMI) values for high school and university samples were 20.91 (standard deviation = 3.04) and 22.47 (standard deviation = 2.63), respectively. Fewer than half (48%) of high school students and two-thirds (66%) of university students rated their overall health as excellent or very good. 

### 3.1. Practicality

The range of response time for high school and university students to complete the questionnaire was 6–17 and 5–15 min, respectively. 

### 3.2. Construct validity 

To ensure clarity, the final draft of the original English version was subjected to five minor revisions as suggested by two expert reviewers. After these revisions, age and income categories were revised. Two Turkish experts reviewed the Turkish translation and suggested two major and seven minor revisions to ensure that the questionnaire met the average literacy level of Turkish high school students and Turkish contextual social acceptability (i.e. cross-cultural validity). The Turkish version was back-translated into English and it was not different from the original English version in terms of content and meaning. To establish face validity, three carefully selected experts individually reviewed each question to ensure the operationalization of each construct against a detailed description of the relevant content domain. Subsequently, three carefully selected Turkish experts individually reviewed the questions, with response options, to ensure that each met the average literacy level of Turkish high school students and Turkish contextual social acceptability (i.e. cross-cultural validity). The survey was revised accordingly and reassessed by all the experts. Prior to utilization, it was also back-translated to English by a professional translator to ensure fidelity with the original concepts.

### 3.3. Reliability

The distribution of ICC estimates between pre- and posttests for questionnaire items was skewed to the left (i.e. towards lower correlations), but the range of ICC estimates was wider for high school students than for university students. The number of items with an ICC value below 0.5 was considerably higher for high school students than for university students, whereas the mean and median ICC values for high school students (0.63 and 0.67, respectively) were lower than those for university students (0.70 and 0.71, respectively). For high school and university, the ICC distribution for each of the five categories is summarized below. 

#### 3.3.1. High school students

The items for the ‘Problem-Public’ category (56 from 15 questions) had a wide range of ICC (0–0.99), with a mean of 0.62 (95% CI = 0.57–0.67), which was significantly greater than the cutoff of 0.5. The items for the ‘Solution-Public’ category (24 from 6 questions) also had a wide range (0–0.95), but the mean ICC, 0.56 (95% CI = 0.46–0.65), was lower compared to the previous category and was not significantly greater than the cutoff. The items for the ‘Problem-Private’ category (6 from 6 questions), compared to both public sphere categories, had a much narrower range of ICC (0.60–0.90) and a noticeably higher mean, 0.76 (95% CI = 0.67–0.85). The items for the ‘Solution-Private’ category (11 from 5 questions), compared to the previous category, had a wider ICC range (0.46–0.99) and a lower mean, 0.70 (95% CI = 0.61–0.80); however, it was still higher than the means for public sphere categories. Finally, the items for the ‘Sociodemographic’ category (4 from 4 questions) had a narrow ICC range (0.68–1) and the highest mean, 0.87 (95% CI = 0.73–0.10). 

#### 3.3.2. University students

Compared to high school students, university students had a narrower ICC range (0.27–0.93) and a greater mean, 0.68 (95% CI = 0.64–0.72), for the ‘Problem-Public’ category. Similarly, the items in the ‘Solution-Public’ category of the university survey, compared to the high school survey, had a narrower ICC range (0.42–0.94) and a much higher mean ICC of 0.75 (95% CI = 0.70–0.80). Unlike for high school students, it was even higher than the mean ICC of the previous category. The items for the ‘Problem-Private’ category had a narrow range of ICC (0.62–0.99), but a markedly higher mean of 0.85 (95% CI = 0.72–0.97) than for high school students. Compared to high school students, university students had a narrower ICC range (0.58–0.96) and a lower mean of 0.64 (95% CI = 0.56–0.73) for the ‘Solution-Private’ category items, which was lower than the means of all three previous categories. Lastly, compared to high school students, the items for the ‘Sociodemographic’ category had a wider ICC range (0.37–0.97) and a much lower mean of 0.60 (95% CI = 0.34–0.87). While not significantly greater than the cutoff, it was the lowest mean of all the categories as well.

## 4. Discussion

To our knowledge, this is the first study that assessed the test-retest reliability of obesity perception items among adolescents and young adults [14]. Overall, the test-retest reliability of items was higher for university students than for school students [29]. In the questionnaire for school students, 26 items that had ICC values of less than 0.5 as well as 36 items with ICC values of 95% CI crossing the 0.5 cutoff (although ICC > 0.5) were disqualified due to poor reliability. However, the number of disqualified questions was markedly lower for university students: only 13 items had ICC values of less than 0.5, whereas 25 items had ICC values of 95% CI crossing the 0.5 cutoff (although ICC > 0.5). The aforementioned items (62 in the high school questionnaire and 38 in the university questionnaire) were deleted. Furthermore, 3 items in the high school questionnaire and 11 items in the university questionnaire were deleted because their retention appeared to be meaningless after the deletion of the aforementioned items [29]. The final questions and items (by category), recommended for high school students (19 questions, 36 items) and university students (26 questions, 52 items), can be found in Table 2 and Table 3, respectively, along with the 95% CI for each item. 

**Table 2:** High school survey’s 19 questions (36 items), selected using test-retest reliability.

How serious a problem is each of these health issues for people in this country: not a problem, only a little serious, moderately serious, very serious, or extremely serious? 1) Cancer, 2) Overweight and obesity, 3) Diabetes, 4) Alcohol/drug abuse, 5) HIV/AIDS
More people are becoming obese these days. These might be causes. For each, please tell if you think it is a major reason, a minor reason, or not a reason for this problem. 1) People spend too much time in front of TV, video games, and computer screens, 2) People do not know how to control their weight, 3) Healthy foods are expensive, 4) People don’t have enough information about what’s in their food, 5) There are not enough safe places for people to be physically active outdoors
Do you think it’s possible for one to be a little overweight and still be healthy? Yes, No
Do you think it’s possible for one to be a lot overweight and still be healthy? Yes, No
How much discrimination do obese people face because of their weight? A lot, a little, some, not very much, or none at all
How many years does obesity shorten an individual’s life expectancy by? <5 years, 5–10 years, 11–15 years, 16–20 years, >21 years
Do you favor the following government policies: Strongly favor, Somewhat favor, Neither favor nor oppose, Somewhat oppose, Strongly oppose? 1) Requiring more physical activity in schools, 2) Requiring restaurants to post calorie information on menus, 3) Limiting the types or amounts of foods and drinks people can buy
How much responsibility does each of the following groups have for solving the country’s obesity problems? A very large amount of responsibility, a large amount, a moderate amount, a small amount of responsibility, or no responsibility at all? 1) Parents and other family members, 2) Food industry, 3) Schools, 4) Health insurance companies, 5) The government, 6) State and local governments,7) Employers
Morbid obesity increases the risk for illnesses like diabetes, high blood pressure, sleep apnea, heart disease, and cancer. Which of the following is the most effective way to treat morbid obesity? 1) Exercise, 2) Diet control, 3) Medication, 4) Surgery, 5) Other
In general, how would you rate your overall health? Excellent, Very good, Good, Fair
Do you personally know anybody who you would consider to be obese? Yes, No
Which of the following best describes your current weight? Underweight, Normal/healthy weight, Overweight, Obese
How do you feel about your current weight? Very happy, Happy, Neither happy nor unhappy, Unhappy, Very unhappy
These questions are about where you live. Is it very easy, somewhat easy, neither easy nor hard, somewhat hard, very hard to… 1) Get to fast food restaurants, 2) Find safe places to be physically active outdoors?
When was your last visit with a doctor for check-up? <6 months ago, 6–12 months ago, 1–2 years ago, >2 years ago
Has your health care provider ever talked with you about the health risks of being or becoming overweight or obese? Yes, No
What is your age? 15–16, 17–18, 19–20
Which one of the following best describes where you live? Urban, Suburban, Rural
Are you male or female? Male, Female

**Table 3:** University survey’s 26 questions (52 items), selected using test-retest reliability.

How serious a problem is each of these health issues for people in this country: not a problem, only a little serious, moderately serious, very serious, or extremely serious? 1) Cancer, 2) Overweight and obesity, 3) Heart disease, 4) Alcohol and drug abuse, 5) Smoking and tobacco use, 6) HIV/AIDS, 7) Mental illness
More people are becoming obese these days. These might be causes. For each, please tell if you think it is a major reason, a minor reason, or not a reason for this problem. 1) People don’t want to change, 2) People don’t know how to control their weight, 3) There is too much unhealthy food, snacks, and drinks for sale in schools, 4) Healthy foods are expensive, 5) People don’t have enough information about what’s in their food, 6) There are not enough safe places for people to be physically active outdoors
Do you think it’s possible for one to be a little overweight and still be healthy? Yes, No
Do you think it’s possible for one to be a lot overweight and still be healthy? Yes, No
How much discrimination do obese people face because of their weight? A lot, a little, some, not very much, or none at all
What is the most serious consequence of being overweight or obese? Heart disease, Diabetes, High blood pressure, Joint problems, High cholesterol, Mental issues, Stroke, Dying young, Cancer, Mobility issues, Respiratory problems, Kidney problems, Other
Which of the following do you think is the greater danger to health? Obesity, Smoking
Which of these do you consider to be the biggest threat to one’s wellbeing, lifestyle, and health, arising from obesity? Tiredness, High blood pressure, Heart disease, Diabetes, Cancer, Sleep apnea, Stroke, Asthma, Low self-esteem and confidence, Depression, Joint and back pain, Limited opportunities for work and career advancement, Other
How many years does obesity shorten an individual’s life expectancy by? <5 years, 5–10 years, 11–15 years, 16–20 years, >21 years?
What should be the government’s involvement in finding solutions to obesity problem? Not involved, slightly involved, moderately involved, very involved, extremely involved
Do you favor the following government policies: Strongly favor, Somewhat favor, Neither favor nor oppose, Somewhat oppose, Strongly oppose? 1) Providing nutritional guidelines and information to people about how to make healthy choices about diet and exercise, 2) Providing incentives to the food industry to produce healthier foods, 3) Requiring restaurants to post calorie information on menus, 4) Banning advertisements for unhealthy foods aimed at children, 5) Placing a tax on the sale of unhealthy foods and drinks, 6) Limiting the types or amounts of foods and drinks people can buy
Which is closer to your opinion? Maintaining a healthy weight is something individuals and families should deal with on their own, It’s something governments, whole communities, schools, healthcare, food industry, etc. need to deal with, or Both
How much responsibility does each of the following groups have for solving the country’s obesity problems? A very large amount of responsibility, a large amount, a moderate amount, a small amount of responsibility, or no responsibility at all? 1) Individual people, 2) Parents and other family members, 3) Food industry, 4) Schools, 5) Health insurance companies, 6) The government, 7) State and local governments, 8) Employers
Is each of these an appropriate treatment for obesity: Never, Almost never, Sometimes, Almost every time, Every time? 1) Exercise, 2) Diet control, 3) Medication, 4) Surgery
Morbid obesity increases the risk of several illnesses. Which of these is the most effective way to treat morbid obesity? Exercise, Diet control, Medication, Surgery, Other
In general, how would you rate your overall health? Excellent, Very good, Good, Fair
About how much do you weigh without shoes? (in kilograms or pounds)
About how tall are you without shoes? (in meters/centimeters or inches/feet)
Do you personally know anybody who you would consider to be obese? Yes, No
Which of these best describes your weight? Underweight, healthy, Overweight, Obese
How do you feel about your current weight? Very happy, Happy, Neither happy nor unhappy, Unhappy, Very unhappy
These questions are about where you live. Is it very easy, somewhat easy, neither easy nor hard, somewhat hard, very hard to ... 1) Find safe places to be physically active outdoors, 2) Buy junk food or fast food when kids are on their way to or from school
When was your last visit with a doctor for check-up? <6 months ago, 6–12 months ago, 1–2 years ago, >2 years ago
Has your health care provider ever talked with you about the health risks of being or becoming overweight or obese? Yes, No
Which of these do you apply first to control your weight? Regular dieting, Counting calories, Regular exercise, Diet pills or supplements, Smoking, Monitoring water intake, Monitoring alcohol intake, Getting enough sleep, Other, None of the above
Are you male or female? Male, Female

Compared to high school students, the test-retest reliability of university students was considerably higher for both public sphere question categories (i.e. problem and solution perception). This is consistent with the fact that the original survey questions were obtained from OPSUS and OPSEU, which were designed for data collection from adults in their respective countries [22,23]. Furthermore, the general understanding is that most adolescents find it difficult to perceive the magnitude of problems at the population level and recommend public policy solutions for consideration. Regardless of the importance of the problem to the general well-being of the country, most adolescents may also not be interested in discussing the problem at all. However, high school students were consistent in perceiving some problems and solutions at the population level, demonstrating their potential to contribute by providing inputs on shaping prevention strategies based on perceived problems, responsibilities, needs, and solutions [14,20]. Their inputs through such surveys would be particularly important in designing health education campaigns and mass media messages for dispelling myths regarding obesity, and also for predicting future trends in public perception of obesity-related problems and solutions [33].

Compared to the public sphere, the private sphere questions had a greater test-retest reliability for high school students, whereas they were more consistent in solution perception in the private sphere compared to university students. These results supported the prior studies which found that children perceive problems and potential solutions in relation to themselves and persons known to them [14]. It is highly likely that their social networks, environments, and experiences affect their perceptions [19,34]. Furthermore, the responses of adolescents to sociodemographic questions were more consistent than the responses of their older counterparts. Higher consistency in these question categories indicated that obesity perception surveys for adolescents could consider collecting more information regarding individual environment, personal experiences, and adolescents themselves [20]. On the other hand, the inconsistency in reporting height and weight by adolescents was in agreement with numerous studies that challenged the reliability of self-reported anthropometric data.

University students were more consistent in responding to problem perception questions and public policy questions, demonstrating their higher knowledge level and ability to express an opinion by processing information received from multiple sources [16]. Furthermore, in contrast to adolescents, young adults were highly reliable in reporting factual details such as height and weight [35]. These findings were somewhat anticipated because the original survey questions addressed persons who were older than 18 years [22,23]. However, university students were relatively inconsistent in solution perception in the private sphere, which may be due to having multiple opinions regarding a given topic. They were also surprisingly inconsistent in reporting sociodemographic information, which may be due to a lack of willingness to disclose actual information. Considering the small sample size, this instrument development study did not intend to compare obesity perceptions among Turkish youth and analogous populations elsewhere, because that will be accomplished in the main study. 

This instrument development study had several limitations. First, the sample size was small; although it was sufficient for the test-reliability analysis [29], it was insufficient for exploratory factor analysis [31]. Second, a common questionnaire was used for both settings (high school and university) intentionally, assuming this approach will be useful in comparing age groups in future surveys; however, the mentioned approach is still open to question [22]. 

In conclusion, the OPSYT developed for high school students consists of 19 questions (36 items), whereas the survey for university students consists of 26 questions (52 items). The OPSYT was the first study that assessed the test-retest reliability of obesity perception items among adolescents and young adults, while the developed instrument has moderate to excellent reliability, with a higher average reliability for young adults. This instrument can be administered longitudinally to suggest changes to policies and interventions, and it can also be utilized after cross-cultural validation to compare obesity perceptions across different populations in the region [29]. 

## Acknowledgments

We thank the European Association for the Study of Obesity, U.K., and the Associated Press-NORC Center for Public Affairs Research, U.S.A., for providing us with their questionnaires.
